# Evaluation of Genetic Diversity and Virulence Potential of *Legionella pneumophila* Isolated from Water Supply Systems of Residential Buildings in Latvia

**DOI:** 10.3390/pathogens12070884

**Published:** 2023-06-28

**Authors:** Olga Valciņa, Daina Pūle, Juris Ķibilds, Linda Labecka, Margarita Terentjeva, Angelika Krūmiņa, Aivars Bērziņš

**Affiliations:** 1 Institute of Food Safety, Animal Health and Environment “BIOR”, LV-1076 Riga, Latvia; olga.valcina@bior.lv (O.V.); daina.pule@bior.lv (D.P.); juris.kibilds@bior.lv (J.Ķ.); linda.labecka@bior.lv (L.L.); 2 Department of Water Engineering and Technology, Riga Technical University, LV-1048 Riga, Latvia; 3 Institute of Food and Environmental Hygiene, Faculty of Veterinary Medicine, Latvia University of Life Sciences and Technologies, LV-3004 Jelgava, Latvia; margarita.terentjeva@lbtu.lv; 4 Department of Infectology, Riga Stradiņš University, LV-1007 Riga, Latvia; angelika.krumina@rsu.lv

**Keywords:** *Legionella*, residential buildings, virulence factors, virulence genes, sequence type

## Abstract

*Legionella* is an opportunistic pathogen with a biphasic life cycle that occasionally infects humans. The aim of the study was to assess the distribution of virulence genes and genetic diversity among *L. pneumophila* isolated from water supply systems of residential buildings in Latvia. In total, 492 water samples from 200 residential buildings were collected. Identification of *Legionella* spp. was performed according to ISO 11731, and 58 isolates were subjected to whole-genome sequencing. At least one *Legionella*-positive sample was found in 112 out of 200 apartment buildings (56.0%). The study revealed extensive sequence-type diversity, where 58 *L. pneumophila* isolates fell into 36 different sequence types. A total of 420 virulence genes were identified, of which 260 genes were found in all sequenced *L. pneumophila* isolates. The virulence genes *enhC, htpB, omp28,* and *mip* were detected in all isolates, suggesting that adhesion, attachment, and entry into host cells are enabled for all isolates. The relative frequency of virulence genes among *L. pneumophila* isolates was high. The high prevalence, extensive genetic diversity, and the wide range of virulence genes indicated that the virulence potential of environmental *Legionella* is high, and proper risk management is of key importance to public health.

## 1. Introduction

*Legionella pneumophila* is a Gram-negative, intracellular bacterial pathogen that causes the potentially fatal but preventable Legionnaires’ disease (LD), which manifests as a severe pneumonia. *L. pneumophila* is also associated with a mild, flu-like illness referred to as Pontiac fever [1]. Although more than 16 serogroups of *L. pneumophila* are known, only half of them have been associated with LD, with the serogroup 1 (SG1) being responsible for the majority of LD cases in Europe. A large proportion of LD cases are diagnosed by a urinary antigen test, which is the best tool for identifying illnesses caused by *L. pneumophila* SG1 [2]. However, widespread application of a urinary antigen test may lead to improper identification and underreporting of other *L. pneumophila* SGs. This may result in delayed epidemiological investigation and implementation of preventive measures against *L. pneumophila* in water circulation systems [3].

*Legionellae* are ubiquitous and have been found in natural and artificial aquatic environments around the world [4]. The range of habitats includes underground and surface bodies of water, moist soils, aquatic plants, and rainforests, although anthropogenic aquatic environments are considered as the main reservoirs. Since *Legionella* can establish biofilms in faucets, shower heads, cooling towers, spas, fountains, and especially in hot water systems, hotpots of *Legionella* infection have been reported in residential buildings, hospitals [5], nursing homes [6], hotels, and in other private and public buildings [7].

The temperature and water stagnation seem to be the most important risk factors for *Legionella* colonization in building water supply systems [8]. The very low counts of *Legionella* characteristic of natural habitats can increase markedly within engineered hot water systems if the recommended temperature regime for the control of opportunistic pathogens has not been followed during routine maintenance in buildings [9]. Contamination of water with *L. pneumophila* in residential buildings has been strongly associated with temperature in the water circulation system [10]. Changes in the water flow may facilitate release of *Legionella* from biofilms localized on the inner surface of pipes in water supply networks, while dead ends with stagnant water or rarely used branches of the pipeline can significantly promote the proliferation of *Legionella*, causing further contamination of water [4,8].

*Legionella* is an opportunistic pathogen with a biphasic life cycle [11]. Like other intracellular bacteria such as *Coxiella*, *Legionella* can alternate between a transmissible (virulent) and a replicative (nonvirulent) phases, which ensure the survival in both nutrient-rich and poor environments and enhance bacterial transfer between different ecological niches [12].

Humans are occasional hosts of *Legionella*, and the ability of the pathogen to infect humans is considered to result from a long coevolution between bacteria and protozoa. The development of LD depends not only on the host’s immunity, bacterial counts, and SGs, but also on virulence factors that the pathogen may contain. The most important virulence factors of *Legionella* are related to the bacterial surface structure and effector protein secretion systems [13]. Surface virulence factors such as the outer membrane proteins, flagella, pili, and lipopolysaccharides promote the initial adhesion to the host cells and enhance infectivity, while toxin production increases the infectivity, survival, and multiplication in alveolar macrophages. Specific genes may regulate biofilm production and increase the ability of the pathogen to survive in water supply systems [14]. Proteins and effectors that provide *Legionella* with their natural ability to infect protozoa also provide the pathogen with the ability to infect mammalian cells. Through horizontal gene transfer from the host amoebae, *Legionella* has acquired numerous eukaryotic-like genes that regulate the infection process [15]. Some of the bacterial effectors can perform parallel functions in different organisms, and some of the effectors can overlap or replace each other in pathogenic and nonpathogenic species [16].

Legionnaires’ disease is an uncommon, sporadic disease with the average incidence of 1.9 cases per 100,000 inhabitants in the EU/EEA in 2020 [2], while 1.4 cases per 100,000 inhabitants were observed in Latvia in the same year. A significant increase occurred in 2021, when 3.2 cases of LD per 100,000 inhabitants were registered [17], which may be explained by greater awareness and more advanced diagnostics.

A multidisciplinary approach to the prevention can be essential for reducing the spread of *L. pneumophila*, hence decreasing the incidence of Legionnaires’ disease. The lack of requirements and preventive actions leads us to assume that in Latvia we can expect a high prevalence and a high genetic diversity of *Legionella* in water supply systems in residential buildings, which can pose a risk to residents. Thus, the aim of the present study was to assess the distribution of virulence genes and genetic diversity among *L. pneumophila* isolated from water supply systems of residential buildings in Latvia.

## 2. Materials and Methods

### 2.1. Sampling

In total, 492 water samples from multistorey residential buildings were collected in sterile bottles, including cold water (*n* = 164) and hot water (*n* = 328). Samples were collected from August 2016 to December 2022 at 200 residential buildings from 26 municipalities in Latvia. For 131 of these residential buildings, there was information available about previous cases of Legionnaires’ disease. There was no such information about the rest (*n* = 69) of the buildings. Buildings that have been linked to LD cases reported in residents relied on thermal disinfection as the primary disinfection method for water supply systems. In buildings without LD history, no additional disinfection or *Legionella* monitoring had been implemented. All the buildings included in the present study were older than 30 years.

Sampling was performed by trained staff in accordance with the requirements of ISO 19458 [18]. At each sampling point, at least one hot water sample was taken from a shower head. Additional samples were taken depending on the size of the building and the responsiveness of the residents and included a cold water sample from a shower head and a hot tap water sample. One liter of water was collected at each location. The water circulation temperature was measured during sampling. A specially equipped vehicle was used to transport the samples to the laboratory while maintaining the temperature between 0 and 6 °C during transportation. Testing of the samples was started no later than 6 h after sampling.

### 2.2. Microbiological Testing of Legionella spp.

Identification and enumeration of *Legionella* spp. was performed according to ISO 11731 [19]. One liter of water was concentrated using a 0.45 μm polyamide membrane filter (Millipore, Molsheim, France). The filter membranes were resuspended in sterile distilled water (5 mL), shaken for two minutes (Vortex Genius, IKA, Staufen, Germany). A total of three 0.1 mL aliquots (untreated, heat-treated, and acid-treated) were spread on Buffered charcoal yeast extract agar (BCYE, Biolife Italiana, Milan, Italy) and Glycine vancomycin polymyxin B cycloheximide agar (GVPC, Biolife Italiana, Milan, Italy). For samples taken before November 2017, only the GVPC medium was used.

The inoculated plates were incubated at 36 °C for 10 days. At least three presumptive colonies of *Legionella* from each plate were subcultured on BCYE agar medium (Biolife Italiana, Milan, Italy) and BCYE agar medium without L-cysteine (BCYE-Cys, Biolife Italiana, Milan, Italy), and incubated at 36 °C for at least 48 h.

Suspected *Legionella* colonies were identified by matrix-assisted laser desorption/ionization time-of-flight mass spectrometry (MALDI-TOF MS, Bruker, Bremen, Germany). An agglutination test (Thermo Fisher Scientific, Bred, The Netherlands) was used for the confirmation of *L. pneumophila*. Individual latex reagents (Pro-Lab Diagnostics, Richmond Hill, Canada) were used for the detection of *L. pneumophila* serogroups.

Presumptive colonies from all BCYE and BCYE-Cys plates were counted and confirmed, and the estimated number of *Legionella* was expressed as CFU/liter with an indication of serogroup. All confirmed *Legionella* isolates were obtained in pure cultures and transferred to the culture collection for long-term storage at −80 °C.

### 2.3. DNA Extraction from Legionella Isolates

From all 197 *L. pneumophila*-positive samples, 58 isolates were selected for genetic analysis. Isolates were selected based on the frequency of *L. pneumophila* serogroup in the analyzed water samples, the geographical origin of the samples, and the year of sampling (Table 1).

The isolates were thawed and cultured on BCYE agar at 37 °C for 48 h before DNA extraction. A single colony from each culture was subjected to manual DNA extraction using the NucleoSpin Tissue reagent kit (Macherey-Nagel, Düren, Germany) according to the manufacturer’s instructions.

### 2.4. Whole-Genome Sequencing

All 58 isolates were subjected to whole-genome sequencing. The wet-lab procedures for library preparation and the bioinformatics procedures for assembly, data quality control, sequence-based typing (SBT), and core-genome MLST (cgMLST) typing were carried out as described previously [20]. Briefly, the trimmed reads were assembled into contigs using the SPAdes assembler v3.14.0 [21]. The contigs were then used as input for further SBT analysis according to the ESCMID *Legionella* Study Group (ESGLI) scheme [22,23] and cgMLST typing according to the scheme published by Moran-Gilad [24]. The collection of core genes among all isolates was determined using the pangenome analysis tool Roary v3.13.0 [25]. The core-gene SNP alignment produced by Roary was then used by FastTree v2.1.10 [26] to build an approximate maximum-likelihood phylogeny.

The virulence factor database (VFDB, retrieved on 12 November 2021) [27] and separately the sequence of the rtxA gene of *L. pneumophila* strain AA100 (nucleotide positions 949-4575 of GenBank ID AF057703.1) were used to identify virulence-encoding genes. The ABricate v1.0.1 tool (https://github.com/tseemann/abricate, accessed on 16 March 2023) was used to screen the assembled genomes for the presence of these virulence genes based on a nucleotide BLAST approach [28]. The thresholds of 80% BLAST sequence identity and 80% length coverage were set to qualify any gene as present in a genome. Additionally, in silico PCR with the rtx1/rtxA-rtx2/rtxA and rtx3/rtxA-rtx4/rtxA primer pairs [29] was simulated using the iPCRess tool from the Exonerate v2.2.0 software package [30]. The presence of antimicrobial resistance (AMR) genes was determined using the ResFinder software v4.1.7 and its associated database (version 2022-05-24) [31]. The same identity and coverage thresholds were used for AMR genes as for virulence genes.

In this study, we selected 11 genes encoding molecules responsible for the ability of *L. pneumophila* to infect humans [16] for further investigation (Table 2).

### 2.5. Data Analysis

R version 4.2.3 (15.03.2023. ucrt), 2023 (The R Foundation for Statistical Computing, Vienna, Austria), package “stats” [43] was used for data analysis. Chi-squared tests and ANOVA were used to calculate differences between the variables. Genotypes were visualized in the form of dendrogram with iTOL v6.7.3 [44] and in the form of minimum spanning tree with GrapeTree v1.5.0 [45].

## 3. Results

### 3.1. Prevalence of L. pneumophila in Residential Buildings

Overall, 197 of 492 (40.0%) samples were *Legionella* spp.-positive (Table 3). Only two *Legionella* species—*L. pneumophila* (*n* = 196) and *L. rubrilucens* (*n* = 1)—were found.

At least one *Legionella*-positive sample was found in 112 out of 200 apartment buildings (56.0%); however, there were no significant differences between buildings linked to known cases of LD (71 positive buildings of 131) and buildings without known LD cases (41 positive buildings of 69; *p* = 0.54).

In buildings with known LD cases, the prevalence of *Legionella* spp. was significantly lower in cold water (*p* < 0.0001), while in buildings without known LD cases, there were no differences observed in the prevalence of *Legionella* between the cold and hot water (*p* = 0.192). Overall, the prevalence of *Legionella* spp. was higher both in cold (*p* < 0.01) and hot water samples in apartment buildings without known LD cases, although for hot water the difference was not statistically significant (*p* = 0.056).

In total, six *L. pneumophila* serogroups (SG) and three combinations of them were identified. Out of all 196 *L. pneumophila*-positive water samples, in 191 cases (97.4%), only one SG was identified. Overall, 55.1% (108/196) of isolates represented SG 2, and 28.1% (55/196) represented SG 3. In five water samples (2.6%), two SGs were found simultaneously (Table 4). No significant differences were observed in the prevalence of SGs in apartment buildings with and without known cases of LD (*p* > 0.05).

The observed levels of *L. pneumophila* colonization varied from 50 CFU/L up to 1.7 × 10^4^ CFU/L, with the average value of 1.8 × 10³ CFU/L. No significant differences were revealed in the levels of colonization with various SGs between buildings with or without known LD cases, except for *L. pneumophila* SG 3, which showed a significantly higher level of colonization (*p* < 0.05) in apartment buildings linked to LD cases (Table 5).

### 3.2. Whole-Genome Sequencing of L. pneumophila

Overall, 36 SBT sequence types (STs) were identified for 58 *L. pneumophila* isolates. Ten SBT sequence types have not been previously documented and are considered as new STs (Table 6).

ST 338 was the most frequent sequence type, which was determined for 10 out of 58 isolates (17%). None of the ST 338 isolates represented *L. pneumophila* SG 1. ST 366 and ST 2002 were detected in three isolates each (5%), while the other STs were detected in no more than two isolates each.

**Figure 1:**
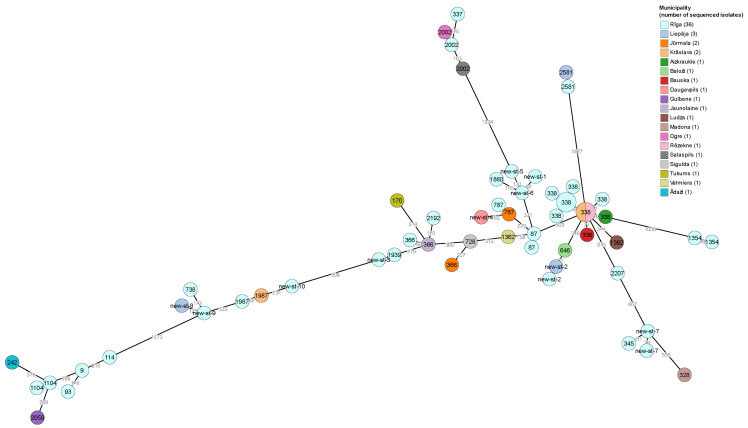
A minimum spanning tree of 58 *L. pneumophila* isolates from water samples taken in apartment buildings in Latvia, based on cgMLST. The node sizes are proportional to the numbers of isolates sharing an identical pattern, while the node colors represent the geographical origin of the isolates, and the node labels indicate STs of the *L. pneumophila* isolates.

According to cgMLST typing, all 58 sequenced *L. pneumophila* isolates fell into 56 different cgMLST types. No cgMLST types specific to geographic location, serogroup, or SBT type were detected. No distinct clusters were identified, but clades around dominant STs such as ST 338 and ST 366 could be discerned, and the clade around ST 1104 was the most distant (Figure 1).

All sequenced *L. pneumophila* isolates had only one antibiotic resistance gene—*aph(9)-la*, encoding the antibiotic resistance factor spectinomycin phosphotransferase.

In total, 420 virulence genes representing 59 gene families were found in 58 sequenced *L. pneumophila* genomes. The number of genes per one isolate varied from 312 to 415 (Table 6), with the average number of 375 virulence genes per isolate. A similar diversity of genes was observed between the isolates from buildings linked to LD cases and buildings without known LD cases. Notable differences between isolates of different serogroups were not found.

The genes *enhC*, *htpB*, *omp28*, *mip*, *mavC*, *legK1*, *sidJ*, *lvhD4*, *lpnE*, *lspC*, and *rtxA* were selected as objects of the greatest interest in this study, and the relative frequency of these genes was evaluated in all 58 isolates (Table 7).

No significant differences were observed in the relative frequency of genes (*p* > 0.05) between buildings linked to LD cases and buildings without known LD cases and between different serogroups, except for *sidJ*, which was the less frequent in SG 9 isolates than in SG 1, SG 2, and SG 3 isolates (*p* < 0.05) and PCR simulated *rtxA*, which was less frequent in SG 1 isolates than in SG 3 isolates (*p* < 0.05).

**Figure 2:**
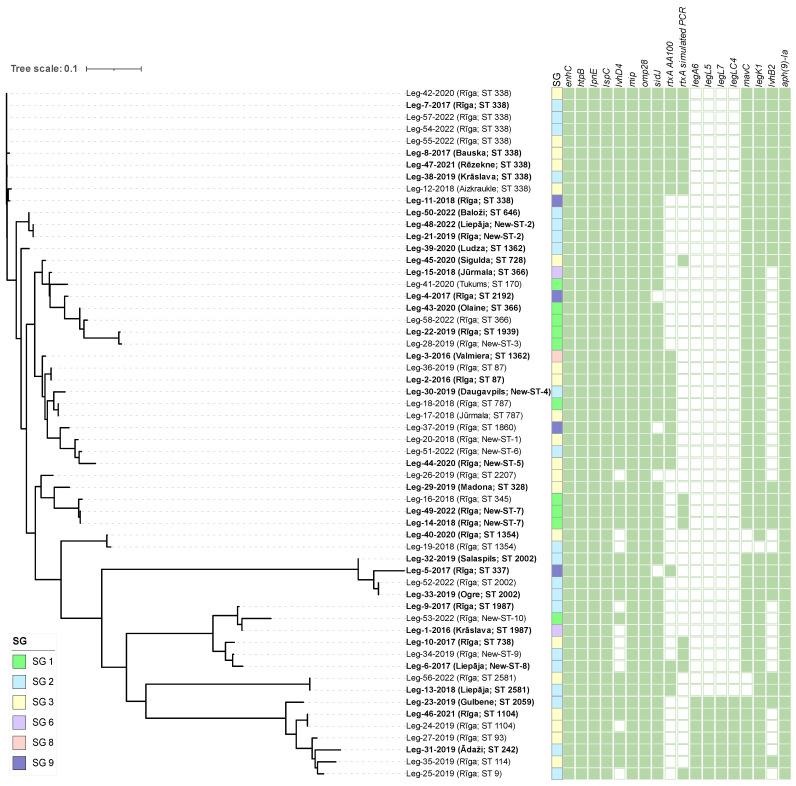
A maximum-likelihood tree built from core-gene alignment of 58 *L. pneumophila* strains. The municipality of the sample origin and SBT type is given in brackets after the name of each node, with strains from buildings linked to LD cases highlighted in bold. Colored squares indicate the serogroup of each isolate. The panel of green squares shows the distribution of *L. pneumophila* virulence and antimicrobial resistance genes among all isolates. Green-filled squares indicate the presence of the gene, while empty squares indicate the absence of the gene in the respective *L. pneumophila* isolate.

A total of 260 genes (62.1%), including *enhC*, *htpB*, *omp28*, *mip*, *lpnE*, and 11 genes of the *lsp* family, were observed in all of the isolates (Figure 2). The Core-Genome SNP dendrogram showed the same clades as the cgMLST minimum spanning tree. The individual leg family virulence genes were found only in seven isolates out of 58 that were present in the clade formed around ST 1104.

The *mav* family was represented by 13 identified genes, of which nine were found in all isolates, while *mavC* was present in 54, *mavG* in 53, *mavH* in 55, and *mavI* in 57 isolates. Altogether, 29 virulence genes of the *leg* family have been identified. The frequency of leg genes in *L. pneumophila* isolates varied from seven to 58. However, only six genes of the *leg* family had a frequency of less than 40: *legU1* was found in 23 isolates, *legC1* in 13 isolates, as well as *legA6*, *legL5*, *legL7*, and *legLC4* in seven isolates each. Out of the 11 virulence genes representing the *sid* family, *sidA*, *sidE*, *sidF*, and *sidK* were found in all 58 isolates. Furthermore, *sidG* and *sidH* were the rarest and were found in six and 13 isolates, respectively. All 11 identified genes of the *lvh* family were found in 46 isolates, except for *lvhB2*, which was found only in 27 isolates.

## 4. Discussion

The evaluation of the presence and diversity of *Legionella* in 200 residential apartment buildings across Latvia revealed high prevalence of *Legionella* in water supply systems of residential buildings. In general, *Legionella* was found in 56% of residential buildings and in 40% of water samples. Our study showed that the prevalence of *L. pneumophila* significantly exceeded the data reported in other studies: 20.7% in Germany [46] and 19.8% in Italy [47]. In the USA, at least one positive *Legionella* sample was found in 15% of single-family homes [48]. However, the prevalence of *L. pneumophila* reported in this study was similar to a previous report from Latvia, where 39% of water samples from apartment buildings were *Legionella*-positive [49]. The high prevalence of *Legionella* in water in Latvia could be related to ineffective water supply system maintenance strategies where water temperature requirements are not met, while water temperature is one of the main factors for *Legionella* persistence and proliferation in building water supply systems [9,10].

Our study showed that water contamination with *Legionella* was found more often (52.3% vs. 35.6%) in samples from buildings with no previous connection to LD cases. The average temperature of hot water was 50.7 °C; however, it was seven degrees lower on average in buildings without previous LD cases. Currently, the temperature of hot water circulation or at the points of consumption is not regulated in Latvia, and the maintenance companies and building managers are obliged to ensure only the temperature at the exit from the heat exchanger, which must not be lower than 55 °C according to the national legislation [50]. Due to the often considerable total length of water circulation pipelines and heat losses in the water supply system between the heat exchanger and the showerheads, the observed hot water temperature at the points of water consumption reached only 45.8 °C in buildings not related to LD cases. In contrast, managers are obliged to carry out disinfection procedures and *Legionella* monitoring in buildings with previous LD history, although guidelines for sampling frequency have not been set. Therefore, in buildings with a previous association with LD, the managers are more aware and, perhaps for this reason, higher hot water temperatures and lower prevalence of culturable *Legionella* were observed in those buildings.

In the present study, six serogroups of *L. pneumophila* were identified, of which SG 2 (55.1%), SG 3 (27.0%), and SG 1 (9.7%) were predominant. However, these results differed from our recent study of *L. pneumophila* in Latvian hotels where SG 3 was the predominant serogroup [20]. It is worth noting that the low prevalence of *L. pneumophila* SG 1 in residential buildings was in agreement with the results from hotels in Latvia [20]. Those results were consistent with low prevalence of *L. pneumophila* SG 1 antibodies (0.2%) in healthy blood donors in Latvia [51]. Living in an apartment building with a centralized hot water supply had been identified as the main environmental risk factor, and the seroprevalence of 9.5% in residents of urban apartment buildings was reported [51]. Globally, *L. pneumophila* SG 1 is considered to be the main causative agent of LD [2] and, accordingly, diagnostic methods for clinical cases have been adapted to identify SG 1. The urine antigen test, which is specific only for *L. pneumophila* SG 1, is still the first-choice method for the diagnostics of LD [2]. Additionally, the incidence of LD could be underreported, because the diagnostics and reports are biased towards SG1; thus, we assume that only the most severe cases, which most likely initially had a higher bacterial load, were detected and reported, while other cases may remain unrecognized. Also, to the best of our knowledge, the absence of clinical isolates of other SGs in Latvia until the end of 2022 may indicate insufficient diagnostics of other SGs.

In five noteworthy cases, two different *L. pneumophila* strains belonging to two different serogroups were found simultaneously in the same water samples. At the time of infection, a person may encounter several *Legionella* strains with different immunological and antibacterial resistance characteristics, thus the choice of appropriate diagnostic methods for clinical cases can present a significant challenge.

Our study showed extensive sequence-type diversity, where 58 *L. pneumophila* isolates fell into 36 different sequence types, 10 of which have not been previously described. It must be admitted that the diversity of sequence types is not unusual for environmental *Legionella*, as confirmed by previous studies from Bosnia and Herzegovina [52], the USA [53], China [54], and Latvia [20]. The higher diversity of STs and the isolation of new STs during the present study could be explained by the focus on residential buildings. In comparison, the lower diversity and lower number of new STs found in hotels during our previous study could be related to internationally distributed STs [20].

The 11 STs found in residential buildings matched those found in Latvian hotels. Moreover, the predominant ST 338 and ST 336 were found both in hotels and in residential buildings. Several STs identified in our study have been associated with LD outbreaks and sporadic cases in other countries [55,56]. In addition, we did not find differences between the STs found in buildings with and without LD cases, and all these findings consequently suggest that *Legionella* strains may persist in residential buildings in Latvia and pose a long-term risk to residents.

In our study, 420 virulence genes were identified, of which 260 genes were found in all sequenced *L. pneumophila* isolates. Genes *enhC*, *htpB*, *omp28*, and *mip* encoding virulence factors related to the bacterial cell surface structures were detected in all isolates, suggesting that adhesion, attachment, and entry into the host cells are enabled for all isolates. The largest group of genes encoding T4SS effectors were quite variable; however, the relative frequency of virulence genes among *L. pneumophila* isolates was high. The wide range of genes encoding effectors demonstrated the high plasticity of the *L. pneumophila* genome and pointed to the possible redundancy of effectors, which is an important feature of *Legionella* [57]. The redundancy within the SidE effector family is well established, where members of the SidE effector family exert the same function on specific host cell targets. SidE, SdeA, SdeB, and SdeC catalyze the ubiquitination of host proteins, and the deletion of all four of these effectors together, but not individually, impairs intracellular growth, which can be restored by the insertion of just one of them [58].

New gene functions in *Legionella* are still being studied, and not all of the *lvh* locus group genes have been assigned a function yet. However, the environment in which *Legionella* grows before contact with the host cell also plays a role. The *lvh* locus gene *lvhB2* is closely related to the ability of the bacterium to infect macrophages or amoebae, depending on the temperature at which *Legionella* grew prior to contact [59].

During the initial analysis, we noticed a remarkable difference in the prevalence of *rtxA*-positive isolates between our study and other studies. The *rtxA* gene was absent in all isolates when screening the genomes against the VFDB database. This was in stark contrast with other studies [60,61], where 20.69–100% of *L. pneumophila* isolates were *rtxA*-positive. However, those studies relied on PCR to determine the presence of this gene. The commonly used *rtx1/rtxA-rtx2/rtxA* and *rtx3/rtxA-rtx4/rtxA* primers have been developed based on the *L. pneumophila* strain AA100 DNA sequence [29], and they only target two approximately 540–630 bp long fragments of the gene. The *rtxA* itself is known to have a modular structure and to be highly variable in length and sequence similarity between different *L. pneumophila* strains [62]. Thereby, we hypothesized that the absence of these two PCR target sites did not necessarily indicate the absence of every possible variant of the *rtxA* gene.

In order to test this hypothesis, we simulated PCR in silico, using the two aforementioned primer pairs and *L. pneumophila* reference sequences that were used to characterize the modular structure of *rtxA* [62]. Only the sequence of strain AA100 produced both in silico PCR products, confirming our hypothesis. Furthermore, the *rtxA* reference (YP_123037) that was included in the respective release of VFDB also did not produce any of the two expected in silico PCR products. Since the *rtxA* sequence from strain AA100 was the shortest of available references and it contained the conserved regions that are located at the start and the end of *rtxA*, we used it as a reference for BLAST-based screening for *rtxA* in our *Legionella* genomes.

We concluded that the method for accurate determination of the presence of *rtxA* should be carefully assessed, taking into account the apparent limitations of either the PCR methods or the alignment-based computational methods and their respective reference databases.

The high prevalence, extensive genetic diversity, and the wide range of virulence genes, which have been detected in all *Legionella* isolates from residential buildings in Latvia, indicate that the virulence potential of environmental *Legionella* is high and that all *Legionella* strains persisting in the water supply systems should be considered as potentially pathogenic. Virulence gene analysis has shown that *Legionella* strains persisting in residential buildings can acquire characteristics and increase the pathogenicity through horizontal gene transfer. Proper risk management, implementation of water safety plans, and microbiological monitoring would ensure the protection of residents by reducing the opportunities for *Legionella* to grow and evolve new virulence traits in man-made water systems.

## Figures and Tables

**Table 1:** The number of *L. pneumophila* isolates from water samples and the number of isolates selected for genetic analysis.

*L. pneumophila* Serogroup	No. of Isolates from Water Samples/ No. of Municipalities of Origin	No. of Selected Isolates/ No. of Municipalities of Origin (%)
SG 1	19/4	9/3 (47/75)
SG 2	108/14	22/11 (20/79)
SG 3	55/10	22/7 (40/70)
SG 6	4/2	2/2 (50/100)
SG 8	1/1	1/1 (100/100)
SG 9	4/1	4/1 (100/100)

**Table 2:** *Legionella* virulence factors.

	Gene	Virulence Factor	Roles
Bacterial surface structures	*enhC*	EnhC	Enhanced entry, trafficking of *Legionella*-containing vacuole [32]
	*htpB*	Hsp60	Attachment, modulation of invasion [33]
	*mip*	MIP	Penetration of the lung epithelial barrier [34]
	*omp28*	MOMP	Mediating phagocytosis [35]
T4SS effectors	*mavC*	MavC	Inhibiting host immunity [36]
	*legK1*	LegK1	Activation of NF-kB [37]
	*sidJ*	SidJ	Calmodulin-activated glutamylase for SidE [38]
	*lvhD4*	VirD4	Coupling protein, reversing virulence defects [39]
	*lpnE*	LpnE	Entry into macrophages and epithelial cells; manipulate host cell trafficking [40]
T2SS effectors	*l* *sp*	Lsp	Transport proteins from the periplasm to the extracellular space [41]
T1SS effectors	*rtxA*	RtxA	Ensures adherence and entry into host and enhances replication, cytotoxicity, and pore forming [42]

**Table 3:** The prevalence of *L. pneumophila* in water samples from residential buildings.

	Buildings with Known LD Cases		Buildings without Known LD Cases		Total	
	Samples Tested/Positive Samples (%)	Average Water Temperature, °C	Samples Tested/Positive Samples (%)	Average Water Temperature, °C	Samples Tested/Positive Samples (%)	Average Water Temperature, °C
Cold water	120/22 (18.3%)	12.9 ± 0.4	44/19 (43.2%)	15.6 ± 0.7	164/41 (25%)	13.5 ± 0.3
Hot water	242/107 (44.2%)	52.1 ± 0.4	86/49 (57.0%)	45.8 ± 1.1	328/156 (47.6%)	50.7 ± 0.4
Total	362/129 (35.6%)	—	130/68 (52.3%)	—	492/197 (40.0%)	—

**Table 4:** The prevalence of *L. pneumophila* serogroups in water samples from buildings with and without previous episodes of LD.

Serogroup	No. of *L. pneumophila* Isolates (%)		
	Buildings with Known LD Cases	Buildings without Known LD Cases	Total
SG 1	15 (11.6%)	4 (6.0%)	19 (9.7%)
SG 2	69 (53.5%)	39 (58.2%)	108 (55.1%)
SG 3	35 (27.1%)	20 (29.9%)	55 (28.1%)
SG 6	3 (2.3%)	1 (1.5%)	4 (2.0%)
SG 8	1 (0.8%)	—	1 (0.5%)
SG 9	2 (1.6%)	2 (3.0%)	4 (2.0%)
SG 3, SG 2	3 (2.3%)	—	3 (1.5%)
SG 3, SG 1	—	1 (1.5%)	1 (0.5%)
SG 3, SG 9	1 (0.8%)	—	1 (0.5%)

**Table 5:** The levels of colonization by *L. pneumophila* in water supply systems.

Serogroup	Levels of *L. pneumophila* Colonization (Min–Max (Average)), CFU/L		
	Buildings with Known LD Cases	Buildings without Known LD Cases	*p*-Value
SG 1	50–4.0 × 10^3^ (8.4 × 10^2^)	4.0 × 10^2^–5.5 × 10^3^ (2.6 × 10^3^)	0.056
SG 2	50–1.3 × 10^3^ (1.5 × 10^3^)	50–6.4 × 10^3^ (2.1 × 10^3^)	0.158
SG 3	50–1.7 × 10^4^ (2.8 × 10^3^)	1.0 × 10^2^–3.9 × 10^3^ (8.2 × 10^2^)	0.033
Total	50–1.7 × 10^4^ (2.0 × 10^3^)	50–6.4 × 10^3^ (1.7 × 10^3^)	0.574

**Table 6:** Characteristics of *L. pneumophila* isolated from water supply systems in Latvia between 2016 and 2022.

Isolate Id	Year of Sampling	Municipality	SG	Linked with LD Cases	Allelic Profile	Sequence Type	Number of Observed Virulence Genes
Leg-1-2016	2016	Krāslava	6	Yes	7,6,17,3,50,11,9	1987	377
Leg-2-2016	2016	Rīga	3	Yes	2,10,3,28,9,4,13	87	372
Leg-3-2016	2016	Valmiera	8	Yes	2,10,3,28,9,4,207	1362	372
Leg-4-2017	2017	Rīga	9	Yes	2,10,24,3,9,4,6	2192	370
Leg-5-2017	2017	Rīga	9	Yes	10,22,7,28,16,18,6	337	358
Leg-6-2017	2017	Liepāja	2	Yes	7,10,17,6,9,11,9	New-ST-8	378
Leg-7-2017	2017	Rīga	2	Yes	2,10,15,28,9,4,13	338	375
Leg-8-2017	2017	Bauska	3	Yes	2,10,15,28,9,4,13	338	378
Leg-9-2017	2017	Rīga	2	Yes	7,6,17,3,50,11,9	1987	378
Leg-10-2017	2017	Rīga	3	Yes	7,10,17,28,17,11,9	738	376
Leg-11-2018	2018	Rīga	9	Yes	2,10,15,28,9,4,13	338	378
Leg-12-2018	2018	Aizkraukle	3	No	2,10,15,28,9,4,13	338	378
Leg-13-2018	2018	Liepāja	2	Yes	2,32,20,38,34,35,219	2581	344
Leg-14-2018	2018	Rīga	1	Yes	6,10,19,3,98,4, novel *neuA* allele	New-ST-7	374
Leg-15-2018	2018	Jūrmala	6	Yes	2,10,3,3,9,4,6	366	374
Leg-16-2018	2018	Rīga	1	No	6,10,19,3,19,4,11	345	374
Leg-17-2018	2018	Jūrmala	3	No	2,10,1,3,9,4,3	787	372
Leg-18-2018	2018	Rīga	1	No	2,10,1,3,9,4,3	787	372
Leg-19-2018	2018	Rīga	2	No	2,10,24,28,4,4,207	1354	312
Leg-20-2018	2018	Rīga	3	No	2,10,3,3,50,4,3	New-ST-1	372
Leg-21-2019	2019	Rīga	2	Yes	2,10,21,28,9,4,6	New-ST-2	374
Leg-22-2019	2019	Rīga	1	Yes	2,10,1,3,9,4,6	1939	374
Leg-23-2019	2019	Gulbene	2	Yes	3,4,1,6,35,9,220	2059	415
Leg-24-2019	2019	Rīga	3	No	3,13,1,28,14,9,13	1104	397
Leg-25-2019	2019	Rīga	2	No	3,10,1,3,14,9,11	9	396
Leg-26-2019	2019	Rīga	3	No	2,10,19,28,19,4,3	2207	360
Leg-27-2019	2019	Rīga	3	No	3,10,1,28,14,9,13	93	415
Leg-28-2019	2019	Rīga	1	No	2,10,17,3,9,4,6	New-ST-3	373
Leg-29-2019	2019	Madona	3	Yes	6,10,19,28,19,4,9	328	375
Leg-30-2019	2019	Daugavpils	2	Yes	2,10,17,3,9,4,9	New-ST-4	369
Leg-31-2019	2019	Ādaži	2	Yes	3,10,1,28,1,9,3	242	415
Leg-32-2019	2019	Salaspils	2	Yes	10,22,7,28,16,18,8	2002	363
Leg-33-2019	2019	Ogre	2	Yes	10,22,7,28,16,18,8	2002	357
Leg-34-2019	2019	Rīga	2	No	7,10,17,6,17,11,9	New-ST-9	377
Leg-35-2019	2019	Rīga	3	No	3,6,1,6,14,11,9	114	415
Leg-36-2019	2019	Rīga	3	No	2,10,3,28,9,4,13	87	373
Leg-37-2019	2019	Rīga	9	No	2,10,3,3,9,4,207	1860	371
Leg-38-2019	2019	Krāslava	2	Yes	2,10,15,28,9,4,13	338	378
Leg-39-2020	2020	Ludza	2	Yes	2,10,3,28,9,4,207	1362	376
Leg-40-2020	2020	Rīga	3	Yes	2,10,24,28,4,4,207	1354	328
Leg-41-2020	2020	Tukums	1	Yes	2,10,3,10,9,4,11	170	375
Leg-42-2020	2020	Rīga	3	No	2,10,15,28,9,4,13	338	371
Leg-43-2020	2020	Olaine	1	Yes	2,10,3,3,9,4,6	366	379
Leg-44-2020	2020	Rīga	3	Yes	2,22,3,28,50,4,3	New-ST-5	371
Leg-45-2020	2020	Sigulda	3	Yes	2,10,3,28,9,4,3	728	373
Leg-46-2021	2021	Rīga	3	Yes	3,13,1,28,14,9,13	1104	412
Leg-47-2021	2021	Rēzekne	3	Yes	2,10,15,28,9,4,13	338	378
Leg-48-2022	2022	Liepāja	2	Yes	2,10,21,28,9,4,6	New-ST-2	372
Leg-49-2022	2022	Rīga	1	Yes	6,10,19,3,17,4,11	New-ST-7	374
Leg-50-2022	2022	Baloži	2	Yes	2,10,21,28,9,4,13	646	375
Leg-51-2022	2022	Rīga	2	No	2,10,3,3,50,4,6	New-ST-6	372
Leg-52-2022	2022	Rīga	2	No	10,22,7,28,16,18,8	2002	360
Leg-53-2022	2022	Rīga	1	No	7,6,17,3,50,4,9	New-ST-10	390
Leg-54-2022	2022	Rīga	2	No	2,10,15,28,9,4,13	338	375
Leg-55-2022	2022	Rīga	3	No	2,10,15,28,9,4,13	338	375
Leg-56-2022	2022	Rīga	3	No	2,32,20,38,34,35,219	2581	344
Leg-57-2022	2022	Rīga	2	No	2,10,15,28,9,4,13	338	375
Leg-58-2022	2022	Rīga	1	No	2,10,3,3,9,4,6	366	375

**Table 7:** The relative frequency of 11 virulence genes in *L. pneumophila* isolates.

Virulence Gene	No. of Positive/No. of Sequenced Isolates	Relative Frequency, %								
		Overall	Isolates from Buildings Linked to LD Cases N = 34	Isolates from Buildings Not Linked to LD Cases N = 24	SG 1 Isolates N = 10	SG 2 Isolates N = 21	SG 3 Isolates N = 20	SG 6 Isolates N = 2	SG 8 Isolates N = 1	SG 9 Isolates N = 4
*enhC*	58/58	100	100	100	100	100	100	100	100	100
*htpB*	58/58	100	100	100	100	100	100	100	100	100
*omp28*	58/58	100	100	100	100	100	100	100	100	100
*mip*	58/58	100	100	100	100	100	100	100	100	100
*mavC*	54/58	93	94	92	100	90	90	100	100	100
*legK1*	57/58	98	100	96	100	95	100	100	100	100
*sidJ*	54/58	93	94	92	100	100	95	100	100	25
*lvhD4*	48/58	83	85	79	100	76	80	50	100	100
*lpnE*	58/58	100	100	100	100	100	100	100	100	100
*lspC*	58/58	100	100	100	100	100	100	100	100	100
*rtxA AA100*	23/58	40	32	50	10	38	55	0	100	50

## Data Availability

The raw FASTQ data obtained from all isolates have been deposited at the European Nucleotide Archive under the project accession number PRJEB61826.
